# Evaluation of tumour infiltrating lymphocytes in luminal breast cancer using artificial intelligence

**DOI:** 10.1038/s41416-023-02451-3

**Published:** 2023-09-30

**Authors:** Shorouk Makhlouf, Noorul Wahab, Michael Toss, Asmaa Ibrahim, Ayat G. Lashen, Nehal M. Atallah, Suzan Ghannam, Mostafa Jahanifar, Wenqi Lu, Simon Graham, Nigel P. Mongan, Mohsin Bilal, Abhir Bhalerao, David Snead, Fayyaz Minhas, Shan E. Ahmed Raza, Nasir Rajpoot, Emad Rakha

**Affiliations:** 1 Academic Unit for Translational Medical Sciences, School of Medicine, University of Nottingham, Nottingham, UK; 2 Department of Pathology, Faculty of Medicine, Assiut University, Assiut, Egypt; 3 Tissue Image Analytics Centre, University of Warwick, Coventry, UK; 4 Department of Histopathology, Sheffield Teaching Hospitals NHS Trust, Sheffield, UK; 5 Department of Pathology, Faculty of Medicine, Suez Canal University, Ismailia, Egypt; 6 Department of Pathology, Faculty of Medicine, Menoufia University, Menoufia, Egypt; 7 Department of Histology and cell biology, Faculty of Medicine, Suez Canal University, Ismailia, Egypt; 8 Biodiscovery Institute, School of Veterinary Medicine and Sciences, University of Nottingham, Nottingham, UK; 9 Department of Pharmacology, Weill Cornell Medicine, New York, NY 10065 USA; 10 University Hospital Coventry and Warwickshire, Coventry, UK; 11 Department of Histopathology, Nottingham University Hospitals NHS Trust, Nottingham, UK; 12 Department of Pathology, Hamad Medical Corporation, Doha, Qatar

**Keywords:** Breast cancer, Lymphocytes

## Abstract

**Background:**

Tumour infiltrating lymphocytes (TILs) are a prognostic parameter in triple-negative and human epidermal growth factor receptor 2 (HER2)-positive breast cancer (BC). However, their role in luminal (oestrogen receptor positive and HER2 negative (ER + /HER2-)) BC remains unclear. In this study, we used artificial intelligence (AI) to assess the prognostic significance of TILs in a large well-characterised cohort of luminal BC.

**Methods:**

Supervised deep learning model analysis of Haematoxylin and Eosin (H&E)-stained whole slide images (WSI) was applied to a cohort of 2231 luminal early-stage BC patients with long-term follow-up. Stromal TILs (sTILs) and intratumoural TILs (tTILs) were quantified and their spatial distribution within tumour tissue, as well as the proportion of stroma involved by sTILs were assessed. The association of TILs with clinicopathological parameters and patient outcome was determined.

**Results:**

A strong positive linear correlation was observed between sTILs and tTILs. High sTILs and tTILs counts, as well as their proximity to stromal and tumour cells (co-occurrence) were associated with poor clinical outcomes and unfavourable clinicopathological parameters including high tumour grade, lymph node metastasis, large tumour size, and young age. AI-based assessment of the proportion of stroma composed of sTILs (as assessed visually in routine practice) was not predictive of patient outcome. tTILs was an independent predictor of worse patient outcome in multivariate Cox Regression analysis.

**Conclusion:**

AI-based detection of TILs counts, and their spatial distribution provides prognostic value in luminal early-stage BC patients. The utilisation of AI algorithms could provide a comprehensive assessment of TILs as a morphological variable in WSIs beyond eyeballing assessment.

## Introduction

Breast cancer (BC) is a heterogeneous disease with different molecular subtypes and variable clinical behaviours [1]. Despite the good prognosis of early-stage BC expressing oestrogen receptor (ER) and lacking human epidermal growth factor receptor 2 (HER2) overexpression (luminal BC), post-treatment recurrence occurs in approximately 20% of cases [2]. This supports the existence of aggressive subgroups within these luminal tumours, which comprise more than 60% of all BC [3]. Therefore, prognostic stratification of early-stage luminal BC is of paramount importance to inform optimal treatment decision-making for these patients [4].

The role of tumour infiltrating lymphocytes (TILs) in refining BC prognosis and possible targeted immunotherapy has been widely studied [5–8]. It has been established that TILs play a key prognostic role in triple-negative (TN) (ER-negative, progesterone receptor (PR)-negative and HER2-negative) and HER2-positive BCs [9]. However, their role in luminal BCs remains unclear and conflicting results have been reported. Some studies reported an association between TILs and poor prognostic parameters [10, 11], while others did not find any prognostic significance [12, 13]. This has led to the exclusion of TILs, as currently assessed, as a prognostic stratifier in luminal BCs [13, 14].

To date, it is recommended that visual assessment of TILs in routine clinical practice depend solely on stromal TILs (sTILs), defined as the percentage of mononuclear cells (lymphocytes and plasma cells) quantifiable in the stromal area [15]. Although this method of assessment would potentially increase reproducibility in routine clinical practice, this approach precludes the assessment of the spatial heterogeneity of TILs distribution which may be clinically informative [15–17]. Similarly, TILs in direct contact with or infiltrating tumour cells known as intra-tumoural TILs (tTILs) are not quantified, despite being biologically relevant to interact with tumour cells [15, 16]. Spatial heterogeneity and tTILs are more challenging to quantify and are considered too complex to visually assess in routine practice.

The widespread use of digital pathology and the increasing applications of artificial intelligence (AI) on whole slide images (WSIs) [18–20] have opened avenues for re-exploring the prognostic roles of morphological features such as TILs, especially within the molecular classes where TILs role is uncertain. The development of machine learning (ML) algorithms for automated computational TILs assessment to allow precise, rapid and less exhaustive workflow is a current need [9]. This would not only improve diagnostic concordance but will also add more information that cannot be assessed by the human eye [21].

In this study, we hypothesised that using AI-based algorithms would provide improved assessment of TILs in early-stage luminal BC patients and identify additional TILs features that have prognostic implications.

## Materials and methods

### Study cohorts

This study was conducted on two cohorts including:

A) Nottingham cohort: a cohort of 2231 endocrine-treated ER + /HER2- BC patients presented to Nottingham City Hospital, Nottingham, United Kingdom. Anonymised clinicopathological data including patient’s age, menopausal status, tumour size, histological subtype, tumour grade, Nottingham prognostic index (NPI), lymph node (LN) status, lymphovascular invasion (LVI), PR and Ki67 expression scores, as well as treatment information were collected. All patients included were treated with adjuvant endocrine therapy only based on local treatment protocol; ER + BC patients were offered chemotherapy only if the NPI score is in the poor prognostic group and the patient tolerates chemotherapy [22, 23]. None of the patients have received neoadjuvant therapy. BC specific survival (BCSS) identified as the time from initial diagnosis to death related to BC was calculated for all patients. The cohort was divided into discovery (*n* = 1572) and test (*n* = 659) sets using stratified random sampling to ensure equal distribution of events in each set. Initially, the discovery set underwent an internal 3-fold cross-validation, where the set was stratified into three different splits which were used for training the model. Then the optimised module resulting from this cross-validation process was applied to the test set. To note, the test set remained untouched throughout the AI model upstream steps to ensure a reliable validation. However, for the simplification of the results, the correlation between TILs against the clinicopathological, and outcome data was carried out on the whole discovery set and validated on the test set.

B) External validation cohort: an external validation cohort (*n* = 318) was also collected from endocrine-treated BC patients presented and managed at the University Hospital Coventry and Warwickshire (UHCW), Coventry, UK from 2011-2014. The clinicopathological data for this cohort was also available.

The patient and tumour characteristics of study cohorts are presented in Supplementary Tables 1 and 2.

Formalin-fixed paraffin-embedded (FFPE) tumour tissue blocks were retrieved for all cases. Fresh sections were prepared and stained with Haematoxylin and Eosin (H&E). Slides were scanned using Philips IntelliSite Ultra-Fast Scanner, Philips Digital Pathology Solutions, Best, the Netherlands and Panoramic 250 Flash III: 3D Histech, Budapest, Hungary on 40x magnification, generating high resolution WSIs. The model was trained on images from both scanners and random brightness/contrast, median blur and colour jitter augmentations were carried out. One representative WSI was used for each case where largest tumour burden with associated TILs was present.

### Annotations of the WSIs

Exhaustive region and cell level annotations were performed by six experienced pathologists for supervised ML training models. Regional annotations involved invasive and in situ tumour areas, tumour associated stroma (TAS), and normal breast tissue (terminal duct lobular units) regions. Cell annotations included tumour cells with various morphologies and degrees of pleomorphism, stromal cells either fibroblasts or myofibroblasts, normal epithelial cells and TILs. Areas of necrosis, calcification and tissue/image artefacts were also annotated and excluded from the final image processing. The detailed process of image acquisition and annotation of this cohort was previously described [24].

### Deep learning pipeline for TILs quantification and distribution

**Fig. 1:**
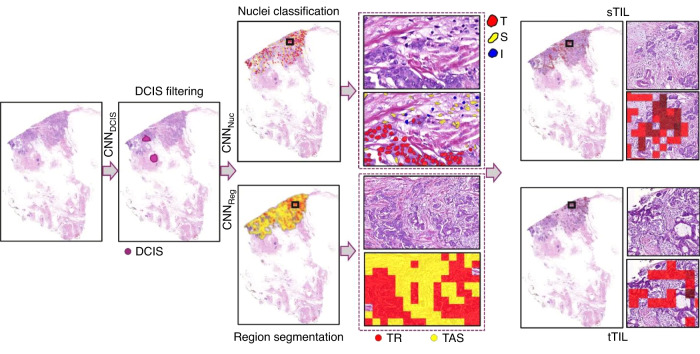
Deep learning-pipeline for generating sTIL and tTIL. Ductal carcinoma in situ (DCIS) is filtered out by CNN_DCIS_ prior to CNN_Nuc_ performs nuclei segmentation and classification followed by CNN_Reg_ performs region segmentation. Both nuclear and regional results are used to generate sTIL and tTIL (T=tumour nuclei, S=stromal nuclei, I=immune nuclei, TR tumour region, TAS tumour associated stroma region).

To quantify sTILs and tTILs, a deep learning (DL) based pipeline is proposed (Fig. 1). Using image thresholding and morphological operations, a tissue mask was generated for a WSI to exclude all irrelevant image background or artefacts from further processing. To exclude regions of carcinoma in situ from the analysis, a convolution neural network (CNN) based model (CNN_DCIS_) [25] was applied. Different types of nuclei including tumour, stromal and normal epithelium were segmented and classified by CNN_Nuc_. CNN_Reg_ was employed to segment stromal and other non-regions of interest (non-ROIs). Further, the stromal regions were restricted to TAS via morphological operations. Finally, the immune, tumour, and stromal nuclei were processed to compute sTILs and tTILs for each WSI.

### Region segmentation

Quantification of immune cells within tumour and TAS requires region segmentation. For the purpose of sematic segmentation of the regions, a CNN model known as the U-Net [26] was adapted by adding two additional encoding/decoding blocks. The model was trained on pathologists’ marked regions and the trained model, referred to as CNN_Reg_, was then used for semantic segmentation of stromal and other non-stromal regions in WSIs. Parameters for training CNN_Reg_ were set as follows: learning rate 0.01 (initial five epochs), 0.001 (epoch 6-10), 0.0001 (epoch 11-30), momentum 0.9, cross entropy loss function, patch size 512 × 512 with 96 pixels context on all sides and batch size 8. The input was normalised to the [0,1] interval and different augmentation methods (random rotate, random brightness/contrast, median blur) were used during training with values of 0.5. To restrict stromal region only to TAS instead of overall stroma, the following steps were performed: i) stromal regions segmented by CNN_Reg_ were combined with tumour regions segmented by CNN_DCIS_; ii) to capture the TAS, the tumour regions were dilated with a disc of radius 8 pixels. This was followed by filling the holes with a disc of radius 32 pixels; iii) any stroma captured in the final dilated tumours was considered as TAS for further feature calculation. Immune nuclei were then counted separately in the tumour regions as well as in the TAS. Other features included co-occurrences of different nuclei, immune heterogeneity, and contrast.

### Nuclei segmentation and classification

**Fig. 2:**
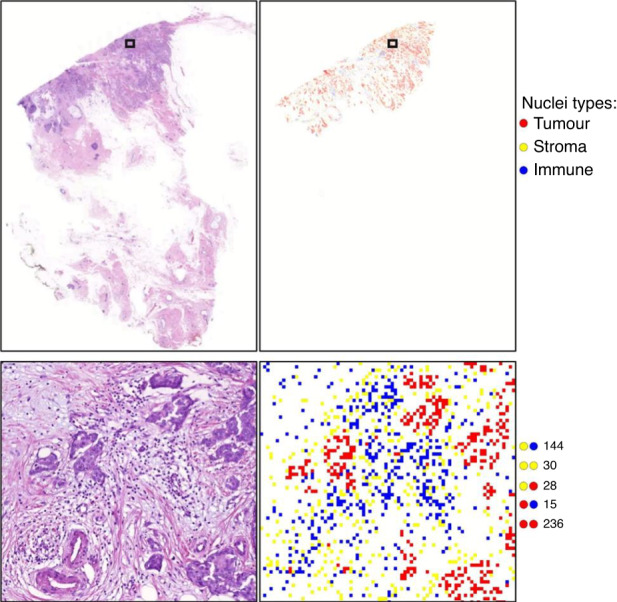
Nuclei segmentation, classification and creation of a co-occurrence matrix. The figure illustrates nuclei segmentation and classification of the tumour, stroma, and immune nuclei (upper) and calculation of the number of times each cell type co-occurs with other cells (lower).

To calculate different features related to immune, tumour and stroma nuclei, an in-house developed state-of-the-art nuclei segmentation and classification model CNN_Nuc_ based on HoVer-Net [27] was fine-tuned to classify and segment different types of nuclei. Weights from a pretrained version of the model on BC only subset of PanNuke dataset [28] were used in the finetuning process. Training parameters were set as follows: learning rate 0.0001 (initial two epochs), 0.00001 (3^rd^ epoch onwards), patch size 256 × 256 pixels and batch size 8. During inference on a WSI, a nuclei mask was generated by mapping the type/class of each detected nucleus at its centroid in a five-times down sampled WSI. Combined with segmented tumour and TAS regions, the counts of immune nuclei were used to get the tTILs and sTILs counts (Fig. 1). Furthermore, a co-occurrence matrix (CM) was created for tumour, stromal and immune nuclei (Fig. 2 and Supplementary Fig. 1). Based on the CM, different features were calculated including immune heterogeneity, stroma and immune co-occurrences, tumour and immune co-occurrences.

### Deep learning-based scoring of sTILs and tTILs

sTILs count refers to the number of sTILs scattered in stroma, while tTILs count describes number of tTILs in direct contact with tumour cells with no intervening stroma [29]. The percentage of tumour stroma occupied by TILs, mimicking the visual score performed by pathologists [15] was added to the features and is referred to as the “AI-based sTILs percentage”. The co-occurrence of sTILs and stromal cells indicates the number of times sTILs and stromal cells are found within certain distance of each other. Similarly, for tTILs, co-occurrence of tTILs with tumour cells within certain distance to each other was also assessed. Supplementary Table 3 illustrates a simplified description of the assessed features.

### Eyeballing assessment of TILs

A subset of cases (*n* = 300) was scored by a well-trained pathologist as a continuous parameter following the proposed scoring method by TILs Working Group [15], where TILs within the tumour borders were only considered, excluding areas of necrosis or tissue artefacts. TILs around normal breast lobules or in situ carcinoma were omitted. Scoring was carried out blindly to the generated features and was then compared with the AI-based sTILs percentage for measuring the concordance as a validation method.

### Statistical analysis

Analysis was performed using Statistical Package SPSS v28 for Windows (Chicago, IL, USA). Assessed features were used as continuous variables and association with clinicopathological parameters was evaluated using Mann-Whitney and Kruskal-Wallis tests. Each feature was dichotomised, into low and high for outcome analysis based on X-tile software version 3.6.1 (Yale University, New Haven, CT) [30] using BCSS as an end point. The cut-off used for analysis was generated on the discovery set and applied to the test set and external validation cohort. Kaplan Meier curves and log-rank test were used to assess the significance between low and high groups and outcome. Cox-regression analysis was performed, hazard ratios (HR) and confidence intervals (CI) were calculated. The power of survival analysis was calculated using Stata Statistical Software, Release 18 (College Station, TX: StataCorp LLC). The level of agreement between visual and AI-based sTILs percentage was assessed using intraclass correlation coefficient (ICC). A *p*-value of less than 0.05 (two-tailed) was considered significant in all the statistical tests.

## Results

### ML modules for TILs quantification and distribution

Analytical performance of different ML modules used in TILs quantification and distribution are shown in Supplementary Table 4. F1-scores for tumour and DCIS segmentation were 0.71 and 0.90, respectively, whereas dice scores of 0.76 and 0.69 were achieved for stromal versus other region segmentation. Similarly, average F1-scores of 0.82, 0.92 and 0.81 were achieved for classifying immune, tumour and stromal cells, respectively. Supplementary Table 5 shows the confusion matrix for cell classification where immune, tumour and stroma cells were classified with accuracies of 80%, 96% and 84.7%, respectively.

### TILs distribution and characteristics

AI-based sTILs percentage had a range from 1% to 76%, which was close to results obtained by visual assessment (ranged from 0 to 80%). The concordance between the visual and AI-based sTILs percentage, showed good agreement (ICC = 0.7).

A significant positive correlation between the numbers of sTILs and tTILs was found (r = 0.6, *p* < 0.001) and this correlation was observed in the discovery and test sets. Similarly, the sTILs count and AI-based sTILs percentage showed significantly positive correlation (r = 0.6, *p* < 0.001) in both sets. In the external validation cohort, the correlation between sTILs and tTILs count and between sTILs count and AI-based sTILs percentage was positive (r = 0.8 and r = 0.6, respectively, *p* < 0.001). The median count of TILs and TILs co-occurrence scores in WSIs, was calculated in the study cohorts (Supplementary Table 3).

### Association of TILs with clinicopathological parameters

High sTILs and tTILs counts as well as the combined total TILs counts were associated with unfavourable clinicopathological prognostic parameters including younger age at diagnosis, larger tumour size, higher grade, LN metastasis, poorer NPI and high Ki67 index (Table 1). Similarly high AI-based sTILs percentage was significantly associated with higher grade, poorer NPI and a high ki67 index (Table 2).

**Table 1:** Association of stromal and intratumoural tumour infiltrating lymphocytes count with clinicopathological parameters in the study cohorts.

	Stromal tumour infiltrating lymphocytes count (sTILs)						Intratumoural tumour infiltrating lymphocytes count (tTILs)					
	Discovery set		Test set		External validation cohort		Discovery set		Test set		External validation cohort	
Characteristics	Mean Rank	*p* value	Mean Rank	*p* value	Mean Rank	*p* value	Mean Rank	*p* value	Mean Rank	*p* value	Mean Rank	*p* value
**Age at diagnosis (years)**												
< 50	925	**<0.001**	366	**0.03**	167	0.6	908	**<0.001**	378	**0.003**	161	0.9
≥ 50	753		323		159		757		320		159	
**Menopausal status**												
Premenopausal	888	**<0.001**	373	**0.003**	NA	NA	868	**<0.001**	372	**0.004**	NA	NA
Postmenopausal	755		319				761		319			
**Tumour size (cm)**												
< 2	706	**<0.001**	293	**<0.001**	145	**<0.001**	719	**<0.001**	302	**<0.001**	141	**<0.001**
≥ 2	1002		415		180		968		393		186	
**Tumour grade**												
1	598	**<0.001**	257	**<0.001**	128	**<0.001**	579	**<0.001**	262	**<0.001**	124	**<0.001**
2	763		328		170		762		325		172	
3	1068		427		212		1091		432		217	
**Mitotic count**												
1	706	**<0.001**	297	**<0.001**	148	**<0.001**	696	**<0.001**	295	**<0.001**	144	**<0.001**
2	944		406		197		942		403		217	
3	1087		433		207		1151		450		211	
**Nuclear pleomorphism**												
1	399	**<0.001**	275	**<0.001**	184	**<0.001**	539	**<0.001**	162	**<0.001**	149	**<0.001**
2	655		277		142		655		290		142	
3	931		394		191		929		382		190	
**Tubule formation**												
1	565	**<0.001**	252	**<0.001**	133	**0.03**	536	**<0.001**	247	**<0.001**	134	**0.02**
2	795		328		158		760		331		156	
3	818		345		171		840		345		173	
**Nottingham Prognostic Index**												
Good prognostic group											138	**<0.001**
Moderate prognostic group	652	**<0.001**	277	**<0.001**	141	**<0.001**	666	**<0.001**	285	**<0.001**	197	
Poor prognostic group	954		394		194		937		386		273	
	1250		458		218		1228		389			
**Histological types**												
No special type (NST)	855	**<0.001**	347	**<0.001**	166	0.05	892	**<0.001**	364	**<0.001**	171	**0.03**
Lobular	673		308		153		602		251		138	
Other special types	475		188		114		397		183		104	
Mixed NST and other tumour types	665		336		152		738		322		157	
**Lymphovascular invasion**												
Negative	759	**<0.001**	320	**<0.001**	154	**<0.001**	764	**<0.001**	326	0.1	155	**<0.001**
Positive	974		399		239		942		357		235	
**Lymph node status**												
Negative	761	**<0.001**	322	**0.04**	156	0.1	767	**<0.001**	324	0.1	156	0.06
Positive	890		360		178		865		354		184	
**Progesterone receptor**												
Negative	838	**0.03**	368	**0.01**	158	0.9	766	0.3	345	0.3	140	0.2
Positive	775		321		160		791		327		161	
**Ki67 index**												
Low (<20%)	300	**<0.001**	140	**<0.001**	NA	NA	300	**<0.001**	140	**<0.001**	NA	NA
High (≥20%)	417		182				417		184			

*NA* not applicable.

Significant *p* values are in bold (Mann-Whitney test).

**Table 2:** Association of artificial intelligence-based stromal TILs percentage with clinicopathological parameters in the study cohorts.

Artificial intelligence-based stromal TILs percentage						
	Discovery set		Test set		External validation cohort	
Characteristics	Mean Rank	*p*-value	Mean Rank	*p*-value	Mean Rank	*p*-value
**Age at diagnosis (years)**						
< 50	845	**0.01**	342	0.4	156	0.8
≥ 50	772		328		160	
**Menopausal status**						
Premenopausal	805	0.3	349	0.2	NA	NA
Postmenopausal	780		325			
**Tumour size (cm)**						
< 2	775	0.1	327	0.5	160	0.9
≥ 2	816		337		159	
**Tumour grade**						
1	569	**<0.001**	266	**<0.001**	147	**0.007**
2	747		329		160	
3	1044		414		207	
**Mitotic count**						
1	717	**<0.001**	302	**<0.001**	154	0.06
2	912		384		170	
3	1054		437		196	
**Nuclear pleomorphism**						
1	704	**<0.001**	302	**<0.001**	184	0.1
2	696		292		154	
3	882		376		171	
**Tubule formation**						
1	575	**<0.001**	257	**<0.001**	134	**0.03**
2	789		319		157	
3	819		349		172	
**Nottingham Prognostic Index**						
Good prognostic group	706	**<0.001**	305	**<0.001**	154	0.3
Moderate prognostic group	886		363		169	
Poor prognostic group	1228		312		184	
**Histological types**						
No special type (NST)	845	**<0.001**	338	0.2	158	0.4
Lobular	650		330		151	
Other special types	645		275		188	
Mixed NST and other tumour types	760		324		164	
**Lymphovascular invasion**						
Negative	767	**<0.001**	323	**0.008**	156	**0.005**
Positive	915		381		217	
**Lymph node status**						
Negative	771	**0.007**	333	0.4	162	0.3
Positive	848		320		146	
**Progesterone receptor**						
Negative	886	**<0.001**	386	**<0.001**	146	0.4
Positive	764		317		161	
**Ki67 index**						
Low (<20%)	300	**<0.001**	140	**<0.001**	NA	NA
High (≥20%)	415		183			

*NA* not applicable

Significant *p* values are in bold (Mann-Whitney test).

The presence of high sTILs-stromal cells co-occurrences, representing the close distribution of TILs to their neighboured stromal cells and similarly tTILs-tumour cells co-occurrences was significantly correlated with unfavourable tumour characteristics in both the discovery and test sets (Table 3). Similar findings were revealed when tested on the external validation cohort (Tables 1–3).

**Table 3:** Association of stromal tumour infiltrating lymphocytes-stromal cells co-occurrence and intratumoural tumour infiltrating lymphocytes-tumour cells co-occurrence with clinicopathological parameters in the study cohorts.

	Stromal tumour infiltrating lymphocytes (sTILs)-stromal cells co-occurrence						Intratumoural tumour infiltrating lymphocytes (tTILs)-tumour cells co-occurrence					
	Discovery set		Test set		External validation cohort		Discovery set		Test set		External validation cohort	
Characteristics	Mean Rank	*p*-value	Mean Rank	*p*-value	Mean Rank	*p*-value	Mean Rank	*p*-value	Mean Rank	*p*-value	Mean Rank	*p*-value
**Age at diagnosis (years)**												
< 50	943	< **0.001**	371	**0.01**	171	0.4	886	< **0.001**	371	**0.01**	160	0.9
≥ 50	749		322		158		751		322		159	
**Menopausal status**												
Premenopausal	906	< **0.001**	380	< **0.001**	NA	NA	849	< **0.001**	364	**0.01**	NA	NA
Postmenopausal	749		317				755		322			
**Tumour size (cm)**												
< 2	721	**<0.001**	298	**<0.001**	147	**0.004**	714	**<0.001**	304	**<0.001**	141	< **0.001**
≥ 2	962		402		177		950		388		186	
**Tumour grade**												
1	611	< **0.001**	265	< **0.001**	128	**<0.001**	569	< **0.001**	262	< **0.001**	123	**<0.001**
2	766		328		170		760		327		173	
3	1045		418		212		1062		424		214	
**Mitotic count**												
1	713	< **0.001**	300	< **0.001**	149	**<0.001**	694	< **0.001**	298	< **0.001**	145	**<0.001**
2	926		401		196		906		393		216	
3	1070		420		202		1129		448		208	
**Nuclear pleomorphism**												
1	365	< **0.001**	255	< **0.001**	188	**<0.001**	545	< **0.001**	158	< **0.001**	157	**<0.001**
2	659		281		141		666		299		144	
3	927		390		192		897		372		187	
**Tubule formation**												
1	590	< **0.001**	261	**0.004**	138	0.07	520	< **0.001**	245	< **0.001**	131	**0.006**
2	794		327		156		732		324		153	
3	814		344		171		842		349		176	
**Nottingham Prognostic Index**												
Good prognostic group	672	< **0.001**	284	< **0.001**	141	**<0.001**	665	< **0.001**	289	< **0.001**	138	**<0.001**
Moderate prognostic group	930		385		194		916		381		198	
Poor prognostic group	1173		450		193		1206		378		271	
**Histological types**												
No special type (NST)	862	< **0.001**	350	< **0.001**	166	**0.002**	873	< **0.001**	362	< **0.001**	169	**0.005**
Lobular	666		298		154		629		262		142	
Other special types	494		194		110		405		195		106	
Mixed NST and other tumour types	750		332		156		721		320		160	
**Lymphovascular invasion**												
Negative	765	< **0.001**	322	**0.003**	155	**<0.001**	758	< **0.001**	329	0.6	155	**<0.001**
Positive	933		388		236		912		338		237	
**Lymph node status**												
Negative	767	< **0.001**	323	0.06	157	0.1	763	**0.009**	326	0.2	155	**0.04**
Positive	866		357		177		837		345		189	
**Progesterone receptor**												
Negative	815	0.2	360	0.05	159	0.9	757	0.3	339	0.5	138	0.2
Positive	780		323		160		782		328		162	
**Ki67 index**												
Low ( < 20%)	301	< **0.001**	140	< **0.001**	NA	NA	297	< **0.001**	140	< **0.001**	NA	NA
High ( ≥ 20%)	414		183				409		182			

*NA* not applicable.

Significant *p*-values are in bold (Mann-Whitney test).

### Outcome analysis

**Fig. 3:**
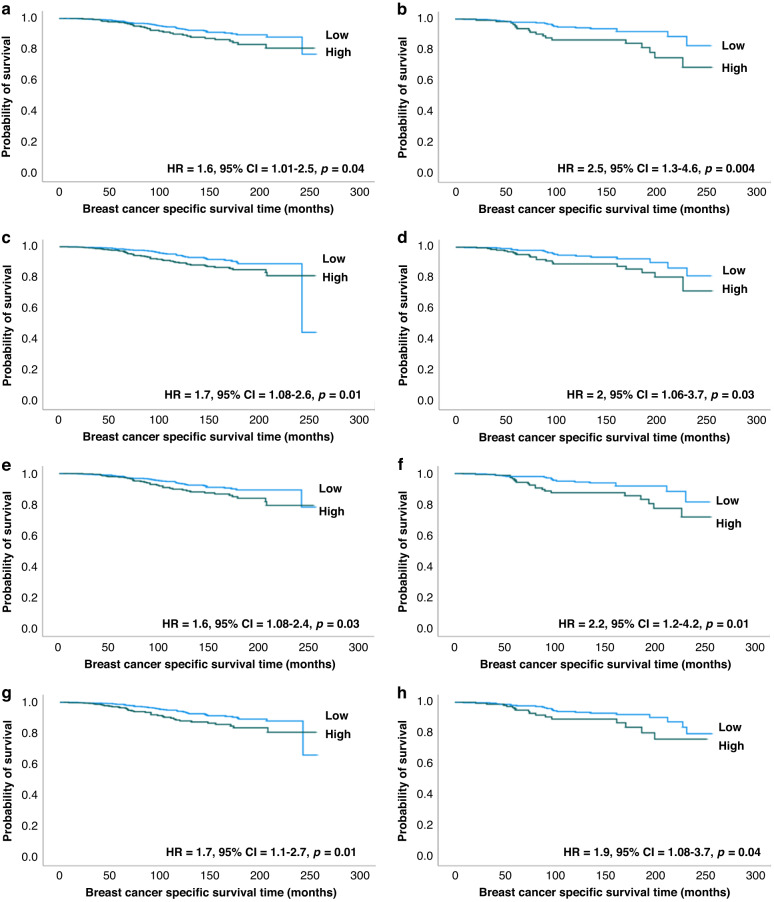
Kaplan Meier curves stratified by assessed tumour infiltrating lymphocytes count and co-occurrence scores. Worse breast cancer specific survival was associated with tumours with high stromal tumour infiltrating lymphocytes count in discovery (**a**) and test (**b**) sets, high intratumoural tumour infiltrating lymphocytes count in discovery (**c**) and test (**d**) sets, high stromal tumour infiltrating lymphocytes- stromal cells co-occurrence in discovery (**e**) and test (**f**) sets, and high intratumoural tumour infiltrating lymphocytes- tumour cells co-occurrence in discovery (**g**) and test (**h**) sets.

Patients with BC showing high sTILs had significantly shorter survival (HR = 1.6, 95% CI = 1.01-2.5, *p* = 0.04, in the discovery set and HR = 2.5, 95% CI = 1.3-4.5, *p* = 0.004 in the test set) than tumours with low sTILs (Fig. 3a, b). The presence of high tTILs was also associated with shorter survival (HR = 1.7, 95% CI = 1.08-2.6, *p* = 0.01 and HR = 2, 95% CI = 1.06-3.7, *p* = 0.03 in discovery and test sets, respectively) (Fig. 3c, d). The same findings were observed using total TILs count. However, sTILs percentage (the current recommended method for TILs assessment) did not show any significant association with patient survival, neither using the AI-based sTILs percentage nor eyeballing scoring (*p* > 0.05).

In terms of the spatial distribution, high sTILs-stromal cells co-occurrence was significantly correlated with worse outcome compared to tumours with low co-occurrence (HR = 1.6, 95% CI = 1.08-2.4, *p* = 0.03 and HR = 2.2, 95% CI = 1.2-4.2, *p* = 0.01 in discovery and test sets, respectively) (Fig. 3e, f). Similar results were observed with high tTILs-tumour cells cooccurrence (HR = 1.7, 95% CI = 1.1-2.7, *p* = 0.01 and HR = 1.9, 95% CI = 1.08-3.7, *p* = 0.04 in discovery and test sets, respectively) (Fig. 3g, h). The power of survival analyses ranged from 60% to 80%.

Multivariate Cox Regression analysis of all significant features, adjusted for tumour grade, LN metastasis, Ki67 index and tumour size, revealed that tTILs count is an independent predictor of outcome. Every 1000 unit increase in tTILs count is associated with 20% increase in BC- related death risk (HR = 1.2, 95% CI = 1.1-1.5 and *p* = 0.007).

However, a statistically meaningful outcome analysis on the external validation cohort could not be carried out due to the limited number of events and the short-term follow-up.

## Discussion

TILs have been extensively studied for their prognostic significance [31–34]. A good prognostic value of TILs in TN and HER2-positive BCs was reported [35–38]. However, TILs findings in luminal ER + /HER2- BCs were inconsistent [10, 12, 39, 40]. Luminal BCs are biologically heterogeneous tumours with divergent clinicopathological characteristics and variable outcomes [12]. A considerable proportion of ER-expressing BCs are resistant to endocrine therapy which highlights the need for further prognostic indicators to stratify high from low-risk early stage luminal BC patients [41].

In the era of immunotherapy, the role of the tumour microenvironment (TME) in predicting patient outcomes, and the possible influence of chemotherapy and hormonal treatment on TME has been demonstrated [42]. Luminal BCs are characterised by low chromosomal instability compared to TNBC, explaining low antigenicity and immune response [43]. Additionally, ER has the capability of T regulators (T-reg) activation and inhibition of antigen-presenting cell (APC) function, promoting immunogenic tolerance [44, 45]. The presence of relatively low TILs levels and studies addressing the absence of prognostic or predictive significance of TILs in ER + /HER2- BCs has shifted attention away from the study of TILs in this patient subgroup [46].

It has been proposed that TILs abundance is sufficient to be assessed in TNBC to guide a prognostic benefit, while in luminal tumours, biological stratification of TILs should be more informative [46, 47]. However, TILs visual evaluation performed by pathologists is limited to a single parameter, stromal TILs [15], which failed to have a prognostic significance in several studies [48, 49]. The TILs working group has recommended assessing only sTILs, taking into account the correspondence with tTILs and the better reproducibility of sTILs between pathologists [13]. Visual assessment cannot capture all complex morphological features and the geometric distribution of TILs in relation to tumour cells which may play a role in BC behaviour. Additionally, low reproducibility and challenges in visual assessment of TILs, highlight the need for reliable automated methods [50, 51]. The agreement in TILs scoring between pathologists has been investigated in several studies with an ICC range from 0.5 to 0.8 [9, 52–54], which emphasises the necessity for a validated standardised method of assessment.

The use of the readily available H&E WSIs in such automated analyses will enable more cost effective use of tissue and clinical resources [17]. Automated TILs detection is expected to enable better identification of TILs features, which are challenging to score by a pathologist owing to their relatively low numbers [47] and difficult recognition among tumour nests [15] and the complexity of assessment of distribution of TILs in relation to stromal and tumour cells. Moreover, computational TILs assessment allows the detection of underestimated parameters in an easy quantitative method, allowing spatial distribution estimation [14]. In the current study, a good level of concordance between visual and automated TILs scores was achieved, which was encouraging for further testing and validation.

Although most previous studies did not focus on tTILs due to their lower density compared to sTILs [40], interestingly, our study revealed that tTILs are an independent prognostic indicator of BCSS, which highlights the under-recognised role of tTILs. In the present study, both sTILs and tTILs were strongly positively correlated, which is consistent with previous studies [55–57], but contrary to Heindl et.al., who proposed that TILs tend to either infiltrate the tumour nests or the stroma [58].

Automated TILs assessment could provide data about the distribution of TILs in relation to stromal and tumour cells. Here the histological ecology of TILs, or how TILs interact with their neighbouring tumour and stromal cells, was investigated in terms of their spatial distribution [59]. It has recently been found that focusing on the spatial relationship may be more predictive than the routinely used density scores [58, 60]. In our study, TILs found in close proximity to tumour and stroma cells were significantly associated with worse clinicopathological features and poorer BCSS, which was consistent with a previous study [58]. However, the co-localisation of tumours and immune cells was reported to have a positive predictive association in luminal A subtype [61]. Their results were regardless of type of therapy received and lacked validation. This controversy encourages further research aiming to study the spatial distribution of TILs, adding to our understanding of tumour progression, managed by the biological interactions between tumour and immune cells [61, 62].

One of the features that can be generated by AI algorithms is the TILs count, which may be a more powerful prognostic indicator than the usual routine assessment method. In relation to patient outcome, TILs count was significantly associated with poorer survival, unlike the sTILs percentage scored both manually and AI-assisted, which lacked prognostic significance. This was consistent with previous studies that reported high TILs were negatively associated with recurrence-free survival and distant metastasis-free survival [2, 63]. It has been reported that high expression of TILs related genes in ER+ BCs was associated with poor overall survival [64]. On the other hand, no prognostic significance of TILs was detected in several studies and meta-analyses [12, 55, 65]. In another AI-based study, none of the TILs abundance scores showed significant association with outcomes, though the discordance may owe to the normalisation to the count of cancer cells [58].

TILs counts were significantly associated with unfavourable clinicopathological parameters in this study in line with the findings of former studies [12, 63]. High TILs in ER + /HER2- BCs have been associated with highly proliferating tumours shown with Ki67 testing which was consistent with several studies [11, 12, 66, 67], that can be explained by increased antigenicity caused by high proliferation. Previous publications reported no additional significance of high TILs in relation to any of the clinicopathological parameters [67, 68], explaining the limited information gained from the visual score.

We propose that one of the contributors to disagreement between studies was the use of different methodologies, whether total TILs or subclasses quantification through IHC detected subpopulations or using different cut-off values for defining low versus high TILs. In our analysis, the TILs count was used as continuous variables to avoid a biased cut-off point.

Our cohort was endocrine treated with poor prognostic outcomes of tumours enriched with TILs. Dunbier and colleagues reported poor response to aromatase inhibitors in ER+ tumours rich TILs suggesting that TILs are involved in resistance to hormonal therapy [69]. Moreover, high TILs was associated with unfavourable outcome in patients who received neoadjuvant letrozole [67]. This suggests that endocrine monotherapy in the subgroup of luminal BC patients with high TILs infiltration is insufficient for optimal patient management and outcomes. Although a clinical trial on immunotherapy for Programmed cell death 1 (PD1)-rich ER + BC patients revealed poor response, neoadjuvant trials had a promising pathological complete response advantage suggesting that ER+ patients encompass a group with possible benefit from immunotherapy [70–72].

We recognise the study has some limitations. Firstly, this study was conducted on H&E-stained sections only which did not allow for the identification of different immune subpopulations and their distinct roles. In routine practice, H&E sections are the standards for TILs evaluation and its association with tumour behaviour and patient outcome, however, the identification of the makeup of the various subpopulations would help in understanding the underlying biology. Various immune cells, including CD8 + , CD4 + , FOXP3 T lymphocytes, B lymphocytes, plasma cells and macrophages (including M1 and M2 subtypes) would have different roles in tumour microenvironment and behaviour. The predominate immune cell type may exert significant control over the tumour behaviour. Secondly, although our model showed high accuracy in identifying and distinguishing TILs from other cell types, we must acknowledge the potential presence of false TILs identification. As with any automated or AI-based system, there is an inherent risk of misclassifications. Thirdly, although using large well characterised cohort, the small number of events in our study warrants careful interpretation of the results and further validation utilising external independent cohorts with long term follow up is recommended.

In conclusion, automated assessment of both TILs counts, and spatial distribution provides independent prognostic value in early-stage luminal BCs. The utilisation of AI algorithms would add to the limited information pathologist can retrieve from visual assessment.

### Supplementary information

Supplementary materials

## Data Availability

The data presented in the current study are available upon reasonable request.
