# Supplementary material for: Evaluation of tumour infiltrating lymphocytes in luminal breast cancer using artificial intelligence
Source: Br J Cancer. 2023 Sep 30;129(11):1747–58. doi: 10.1038/s41416-023-02451-3 (PMC10667537; doi:10.1038/s41416-023-02451-3)
Supplement: Supplementary file 1 — Supplementary materials [file 41416_2023_2451_MOESM1_ESM.docx]

**Supplementary Table 1:** Patient and tumour characteristics of Nottingham cohort.

| **Characteristics** | **Discovery set**  (n=1,572) | **Test set**  (n=659) | ***X*^2^**  **(*p*-value)** |
| --- | --- | --- | --- |
|  | **Frequency (%)** | **Frequency (%)** |  |
| **Age at diagnosis (Years)**  < 50  ≥ 50 | 303 (19)  1269 (81) | 110 (17)  549 (83) | 2  (0.1) |
| **Menopausal status**  Premenopausal  Postmenopausal | 377 (24)  1195 (76) | 135 (20)  524 (80) | 3.2  (0.07) |
| **Tumour size (cm)**  < 2  ≥ 2 | 1146 (73)  426 (27) | 458 (69)  201 (31) | 2.7  (0.1) |
| **Tumour grade**  1  2  3 | 350 (22)  912 (58)  310 (20) | 159 (24)  373 (57)  127 (19) | 0.9  (0.6) |
| **Mitotic count**  1  2  3 | 1156 (74)  226 (14)  190 (12) | 478 (72)  109 (17)  72 (11) | 2  (0.3) |
| **Nuclear pleomorphism**  1  2  3 | 10 (1)  804 (51)  758 (48) | 9 (1)  351 (53)  299 (46) | 4  (0.1) |
| **Tubule formation**  1  2  3 | 151 (10)  479 (30)  942 (60) | 70 (10)  202 (31)  387 (59) | 0.6  (0.7) |
| **Nottingham Prognostic Index**  Good prognostic group  Moderate prognostic group  Poor prognostic group | 906 (58)  633 (40)  33 (2) | 367 (56)  281 (43)  11 (1) | 1.4  (0.5) |
| **Histological types**  No special type (NST)  Lobular  Other special types  Mixed NST and other tumour types | 887 (56)  217 (14)  91 (6)  377 (24) | 387 (59)  79 (12)  40 (6)  153 (23) | 1.8  (0.6) |
| **Lymphovascular invasion**  Negative  Positive | 1378 (87)  200 (13) | 575 (87)  84 (13) | 0.0001  (0.9) |
| **Lymph node status**  Negative  Positive | 1261 (80)  311 (20) | 525 (80)  134 (20) | 0.08  (0.7) |
| **Progesterone receptor**  Negative  Positive | 284 (18)  1288 (82) | 121 (18)  538 (82) | 0.03  (0.8) |
| **Ki67 index**  Low (<20%)  High (≥20%) | 478 (72)  186 (28) | 227 (76)  73 (24) | 3.5  (0.1) |

**Supplementary Table 2:** Patient and tumour characteristics of the external validation cohort.

| **Characteristics** | **Frequency (%)** |
| --- | --- |
| **Age at diagnosis (Years)**  < 50  ≥ 50 | 36 (11)  282 (89) |
| **Tumour size (cm)**  < 2  ≥ 2 | 187 (59)  131 (41) |
| **Tumour grade**  1  2  3 | 109 (34)  180 (57)  29 (9) |
| **Mitotic count**  1  2  3 | 249 (78)  43 (14)  26 (8) |
| **Nuclear pleomorphism**  1  2  3 | 2 (1)  204 (64)  112 (35) |
| **Tubule formation**  1  2  3 | 52 (16)  130 (41)  136 (43) |
| **Nottingham Prognostic Index**  Good prognostic group  Moderate prognostic group  Poor prognostic group | 208 (65)  107 (34)  3 (1) |
| **Histological types**  No special type (NST)  Lobular  Other special types  Mixed NST and other tumour types | 226 (71)  50 (16)  25 (8)  17 (5) |
| **Lymphovascular invasion**  Negative  Positive | 299 (94)  19 (6) |
| **Lymph node status**  Negative  Positive | 274 (86)  44 (14) |
| **Progesterone receptor**  Negative  Positive | 28 (9)  290 (91) |

**Supplementary Table 3**. Description of calculated scores used for tumour infiltrating lymphocytes (TILs) assessment.

| **Scores** | **Description** | **Discovery set**  **Mean Median** | **Test set**  **Mean Median** | **External validation cohort**  **Mean Median** |
| --- | --- | --- | --- | --- |
| **Number of stromal TILs** | Number of TILs in tumour stroma; scattered in stroma between tumour cells | 7795 3346 | 7196 3346 | 4071 1769 |
| **Number of intratumoural TILs** | Number of intratumoural TILs; in direct contact with tumour cells | 4215 1267 | 2653 1235 | 2143 725 |
| **Artificial intelligence (AI) based stromal TILs percentage** | Percentage of tumour stroma occupied by TILs | 12% 9% | 12% 9% | 9% 7% |
| **Stromal TILs-stromal cells co-occurrence** | Number of times stromal TILs and stromal cells are found within certain distance to each other | 2935 1560 | 2653 1650 | 2124 808 |
| **Intratumoural TILs-tumour cells co-occurrence** | Number of times tumoural TILs and tumour cells are found within certain distance to each other | 3203 1173 | 2838 1128 | 2560 766 |

**Supplementary Table 4.** Performance of different machine learning modules used in the proposed pipeline.

| **Module** | **Metric** | | | **Subset** |
| --- | --- | --- | --- | --- |
| **DCIS filtering** | **F1-score** | | | 5-fold cross-validation on discovery set |
|  | Tumour | DCIS | |  |
|  | 0.71±0.03 | 0.90±0.01 | |  |
| **Region segmentation** | **Dice** | | | Test set |
|  | Stroma | Other | |  |
|  | 0.76 | 0.69 | |  |
| **Nuclei classification** | **F1-score** | | | 3-fold cross-validation on 29 cases from the discovery set |
|  | Immune | Tumour | Stroma |  |
|  | 0.82±0.06 | 0.92±0.02 | 0.81±0.03 |  |

**Supplementary Table 5.** Confusion matrix for cell classification module of the proposed pipeline.

|  | **Immune** | **Tumour** | **Stroma** |
| --- | --- | --- | --- |
| **Immune** | 4083 (80%) | 445 (8.6%) | 584 (11.4%) |
| **Tumour** | 166 (0.9%) | 16841 (96%) | 547 (3.1%) |
| **Stroma** | 525 (5.5%) | 941 (9.8%) | 8092 (84.7%) |


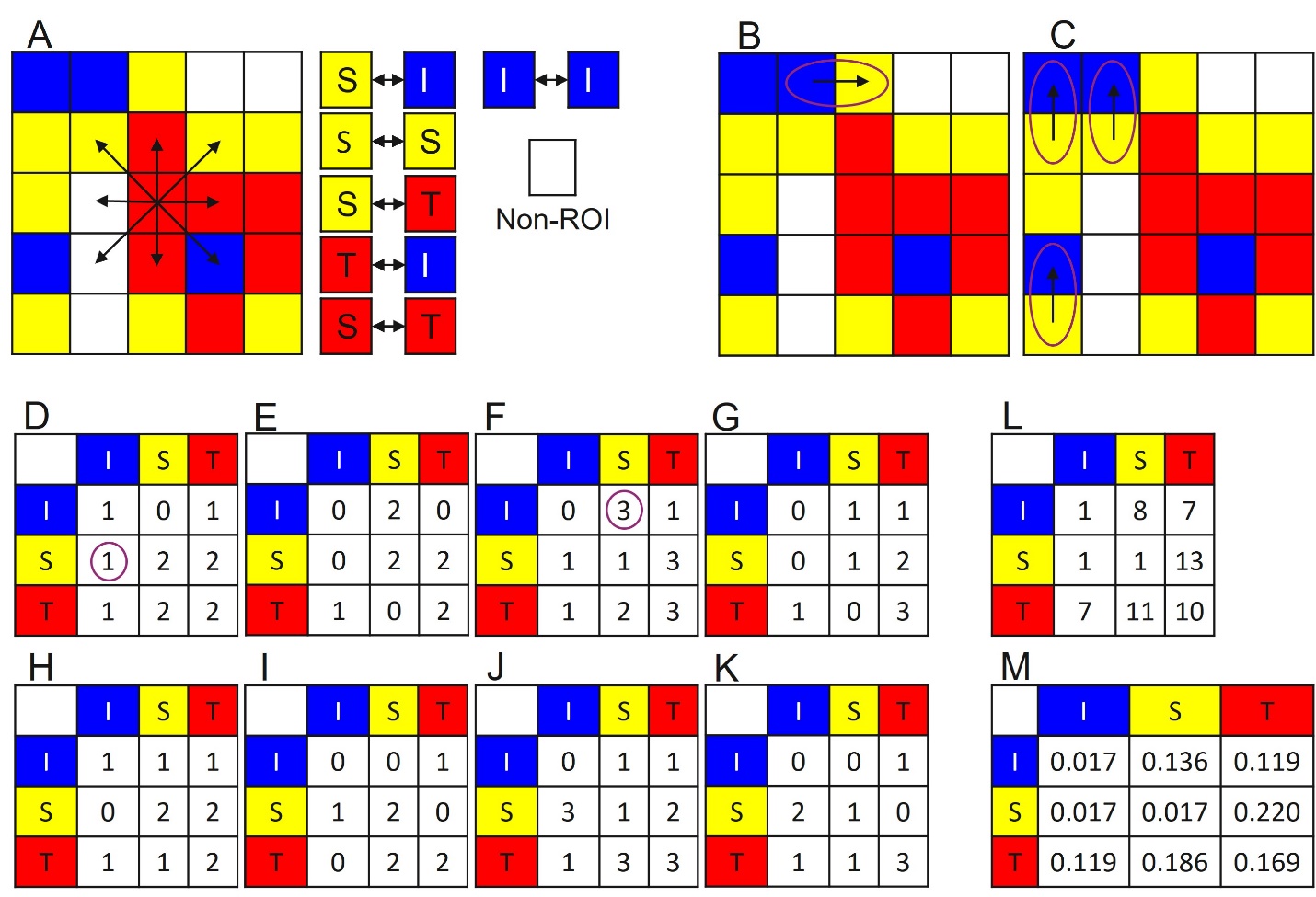


**Supplementary Figure 1.** The number of times predicted immune (I), stroma (S) and tumour nuclei neighbour with each other at a distance of one cell hop on any of the 8-direction is shown **(A)**. S occurring to the right (i.e., at angle 0) of I is 1 and is shown by an ellipse in **B** (and circled in **D**). Similarly, I occurring above (i.e., at angle 90) S is 3 and is shown by ellipses in **C** (and circled in **F**). Such occurrences can be calculated at 8 different angles (0, 45, 90, 135, 180, 225, 270, and 315 shown in **D, E, F, G, H, I, J** and **K**, respectively) and then summed to get total occurrences **(L)**. Note: to get the sum of occurrences of the same entity for example T with T, the total occurrences are divided by 2. The resulting values are then normalised (i.e., each value in the table is divided by the total sum of the values) and the resulting table – termed Co-occurrence Matrix (CM) **(M)** is then used to calculate different features such as contrast and heterogeneity.
